# Morphological and phylogenetic features of the Crimean population of Juniperus deltoides R.P. Adams

**DOI:** 10.18699/VJGB-23-37

**Published:** 2023-07

**Authors:** A.O. Lantushenko, O.O. Korenkova, A.A. Syrovets, Ya.V. Meger, P.A. Korenkov, O.M. Shevchuk

**Affiliations:** Sevastopol State University, Sevastopol, Russia; The Order of the Red Banner of Labour Nikitsky Botanical Gardens – National Scientific Center of the Russian Academy of Sciences, Yalta, Republik of the Crimea, Russia; Sevastopol State University, Sevastopol, Russia; Sevastopol State University, Sevastopol, Russia; Moscow State University of Civil Engineering (National Research University), Moscow, Russia; The Order of the Red Banner of Labour Nikitsky Botanical Gardens – National Scientific Center of the Russian Academy of Sciences, Yalta, Republik of the Crimea, Russia

**Keywords:** Juniperus deltoids, population, needles, cone berries, cryptic view, phylogenetic analysis, nuclear genes, chloroplast genes, Juniperus deltoids, популяция, хвоя, шишкоягоды, криптический вид, филогенетический анализ, ядерные гены, хлоропластные гены

## Abstract

Juniperus deltoides is a relict species from the Tertiary Period. It is a typical representative of the Mediterranean group of the section Juniperus. It is included in the Red Books of the Republic of Crimea and the city of Sevastopol. Until recently, it was believed that a population of J. oxycedrus grew in Crimea. Currently, J. deltoides is described as a cryptic species, morphologically difficult to distinguish from J. oxycedrus. As a result, it became necessary to conduct a series of detailed studies to determine the morphological and phylogenetic features of the Crimean cryptic population in order to identify it as being one of the species of the cryptic pair. The studies were carried out in two stages: at the first stage, the morphological features of the vegetative and generative organs and their difference from J. oxycedrus were determined; the second stage included genetic research. The length of the needles of the Crimean population is 12.94 ± 0.19 mm, which corresponds to the Eastern Italian population of J. deltoides. At the same time, the width of the needles is 1.39 ± 0.02 mm, which is typical of the Portuguese population of J. oxycedrus. The dimensions of the cones are d1 (conditional height) = 7.54 ± 0.14 mm, and d2 (conditional width) = 9.11 ± 0.09 mm, which is more in line with J. deltoides. The shapes of the cones are very diverse. Some individuals have cones, the covering scales of which are visually indistinguishable, and their tops are completely fused. A similar phenomenon is characteristic of the Western Mediterranean populations of J. oxycedrus. Morphological analysis of the vegetative and generative organs of J. deltoides showed that when these two traits are combined, it is not possible to reliably distinguish between J. deltoides and J. oxycedrus individuals. Nuclear (ITS internal transcribed spacer) and chloroplast (petN-psbM, trnS-trnG) non-coding regions of the genome were used for genetic analysis. Studies have shown that the nuclear regions of genes have greater variability than chloroplast regions. The sequences obtained in this work formed a clade with J. deltoides samples 9430 and 9431 (BAYLU) growing in Turkey, which makes it possible to assign the samples studied to J. deltoides.

## Abstract (ru)

Juniperus deltoides – это реликтовый вид третичного периода. Является типичным представителем средиземноморской группы секции Juniperus. Включен в Красные книги Республики Крым и города Севастополя. До недавнего времени считалось, что на территории Крыма произрастает популяция J. oxycedrus. Сейчас J. deltoides описывают как криптический вид, морфологически сложно отличимый от J. oxycedrus. В результате возникла необходимость проведения ряда детальных исследований по определению морфологических и филогенетических особенностей криптической популяции Крыма с целью установления ее принадлежности к одному из видов криптической пары. Исследования проходили в два этапа: на первом этапе определяли морфологические особенности вегетативных и генеративных органов и их отличие от J. oxycedrus, на втором – выполняли генетические исследования. Длина хвои крымской популяции составляет 12.94 ± 0.19 мм, что соответствует восточноитальянской популяции J. deltoides. При этом ширина хвои равна 1.39 ± 0.02 мм, что свойственно португальской популяции J. oxycedrus. Размеры шишкоягод составляют: d1 (условно высота) – 7.54 ± 0.14 мм, d2 (условно ширина) – 9.11 ± 0.09 мм, что больше соответствует J. deltoides. Форма шишкоягод варьирует весьма сильно. Встречаются особи с шишкоягодами, кроющие чешуи которых визуально не отличимы, их верхушки полностью срастаются. Подобное явление характерно для западносредиземноморских по- пуляций J. oxycedrus. Морфологический анализ вегетативных и генеративных органов J. deltoides показал, что при сочетании двух этих признаков достоверно различить особи J. deltoides и J. oxycedrus не представляется возможным. Для генетического анализа использовали ядерные (внутренний транскрибируемый спейсер ITS) и хлоропластные (petN-psbM, trnS-trnG) некодирующие участки генома. Исследования показали, что ядерные участки генов обладают большей вариабельностью, чем хлоропластные. Последовательности, полученные в данной работе, образовали кладу с образцами J. deltoides 9430 и 9431 (BAYLU), произрастающими в Турции, что позволяет отнести изученные образцы к виду J. deltoides

## Introduction

The genus Juniperus L. is the largest in the cypress family (Cupressaceae Bartl.), assigned to the juniper subfamily (Juniperoideae Endl.), and includes 76 species (Pisarev, 2007; Adams, 2014b).

The genus Juniperus L. is the largest in the cypress family (Cupressaceae Bartl.), assigned to the juniper subfamily (Juniperoideae Endl.), and includes 76 species (Pisarev, 2007; Adams, 2014b).

The genus was first described in 1700; since that time, the taxonomy of the genus has undergone significant changes (Novikov et al., 2014). Currently, the genus is divided into three sections, among which: the Caryocedrus section counting one species – J. drupacea Labill., the Juniperus section (synonymous with Oxycedrus), which includes 14 species, and the Sabina section, which consists of the remaining 61 species (Pisarev, 2007; Abaimov, 2009; Adams, 2014b). At the same time, the Caryocedrus section was often considered as a separate genus, but PCR studies conducted by R.P. Adams proved its common origin with the Juniperus section (Adams, 2014b).

Five species of junipers grow in the Crimea (J. communis L., J. deltoides R.P. Adams, J. excelsa M.-Bieb., J. foetidissima Willd., J. sabina L.), which belong to two sections – Juniperus and Sabina. All of them are included in the Red Books of the Republic of Crimea and the city of Sevastopol (Yena, Fateryga, 2015; Red Book…, 2018).

Juniperus deltoides is a relict species from the Tertiary period. It is a typical representative of the Mediterranean group of the Juniperus section. J. deltoides is common in the Mediterranean and the Middle East. To a large extent, its range is limited to the Mediterranean climate, but in the Balkans it occurs in more continental conditions. The northern border of its range passes in the Crimea. The area of the cryptic population of the Crimea, according to 2006 data, is 4843 hectares (Adams, 2014b; Plugatar, 2015; Farjon, 2017; Rajčević et al., 2020; Sadykova, Neshataeva, 2020; Yousefi et al., 2021).

Until recently, it was believed that a population of J. oxycedrus grows on the territory of Crimea. This species was included in the Guide to Higher Plants of Crimea (Rubtsov, 1972). However, the scientist R.P. Adams, on the basis of DNA sequencing, found that in most of the Mediterranean – from Italy to the east through Turkey to the mountains of the Caucasus and Iran (including the Crimea), a juniper other than J. oxycedrus is common, which he described as a new species – J. deltoides. At the same time, no direct analysis of the genetic material from the territory of Crimea was carried out. Adams made a similar conclusion based on the geographic localization of populations. In his works, he described that junipers growing west of Italy belong to the species J. oxycedrus, and junipers found to the east are J. deltoides (Adams et al., 2005; Adams, 2014a; Roma-Marzio et al., 2017).

Currently, J. deltoides is described as a cryptic species that is morphologically difficult to distinguish from J. oxycedrus (Adams et al., 2005; Adams, 2014a; Roma-Marzio et al., 2017). As a result, it became necessary to conduct a series of detailed studies to determine the morphological and phylogenetic features of the Crimean population of J. deltoides, in order to establish its belonging to one of the species of the cryptic pair.

The study includes two main tasks: determining the correspondence between the morphological features of the vegetative and generative organs of the cryptic population of the Crimea to the species J. deltoides; conducting genetic studies using nuclear and chloroplast regions of marker sequences.

## Materials and methods

**Fig. 1.**
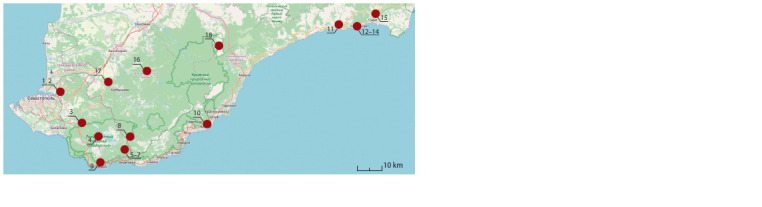
Scheme of the location of sample plots in the cryptic populations of the Crimean Mountains. 1, 2 – the vicinity of the city of Inkerman; 3 – mt. Chirka-Kayasy; 4 – mt. Samnalykh; 5–7 – mt. Kara-Dag; 8 – mt. Tolaka- Bair; 9 – mt. Dragon; 10 – Cape Martyan; 11 – mt. Papaya-Kaya; 12, 13 – mt. Koba-Kaya; 14 – mt. Sokol; 15 – mt. Karshiters; 16 – Kullu-Kaya rocks; 17 – the vicinity of the village of Kudrino; 18 – mt. Chatyr-Dag.

In order to conduct morphological and phylogenetic studies within the population, test areas of 0.2 hectares were laid at an altitude of 40 to 620 m above sea level, in various edaphoorographic conditions from Inkerman to Sudak (Fig. 1).

According to generally accepted methods, 10 model trees were identified within the test areas (Yarmishko, Lyanguzova, 2002). For each model tree, 30 cones were measured in two mutually perpendicular planes (conventional width and height). In addition, according to the determinant key developed by Adams for J. deltoides (Adams, 2014a), the degree of accretion of cone scales was visually determined.

To determine the parameters of the vegetative organs, the length and width of the needles were measured and the average error was determined (30 needles for each model tree). Then, a cross section of the needles was carried out in order to establish the presence or absence of curvature of the adaxial surface of the needles. The shape of the base of the needles was determined (Adams, 2014a, b).

18 samples of J. deltoides from different geographical locations of the Crimean peninsula were selected for genetic studies (see Fig. 1). DNA isolation from needles was carried out using the DNeasy Plant Mini Kit (Qiagen, Germany). The quantity and quality of the isolated DNA were analyzed using an Inplen nanophotometer (Germany). For PCR analysis, nuclear (internal transcribed spacer ITS) and chloroplast (petN-psbM, trnS-trnG) non-coding regions of the genome were used. Marker gene amplifications were performed using the universal primers and protocols described earlier (Table 1, Hojjati et al., 2018), using the ScreenMix reagent kit (Eurogen, Russia).

**Table 1.**
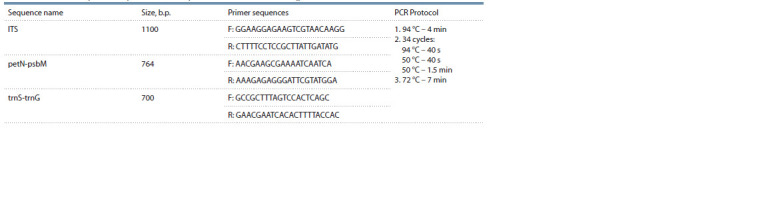
Nucleotide sequences of primers and PCR protocol used in this work (Hojjati et al., 2018)

Sequencing of the obtained fragments was carried out on the genetic analyzer NANOPHOR-05 (Syntol, Russia) in the Resource Centre “Molecular Structure of Matter”. Electrophoregrams unsuitable for analysis were obtained for two samples during sequencing of the ITS nuclear fragment, and the nucleotide sequences of 16 samples of the Crimean population were further studied (Table 2). The obtained sequences of ITS, petN-psbM and trnS-trnG were compared with those available in the database of the National Center for Biotechnology Information (https://www.ncbi.nlm.nih. gov/). The samples for comparison were taken from (Hojjati et al., 2018) and are indicated in Table 2. The alignment of nucleotide sequences for each marker site and their integration into the combined matrix was carried out in the MegaX program (Kumar et al., 2018). Phylogenetic reconstruction was performed using the Bayesian method implemented in MrBayes version 3.2.6 (Ronquist et al., 2012).

**Table 2.**
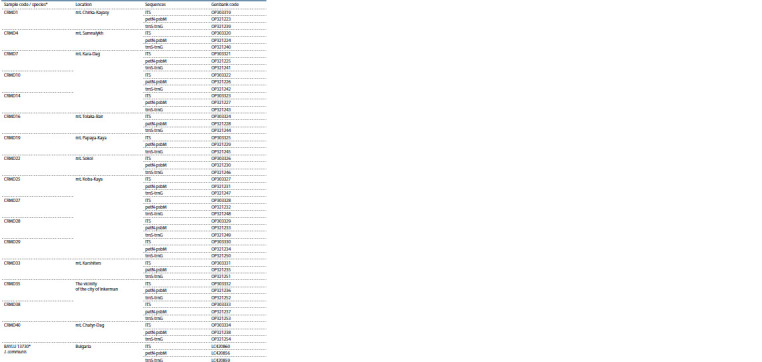
Codes, geographical location and Genbank codes of the analyzed samples

**Table 2end.**
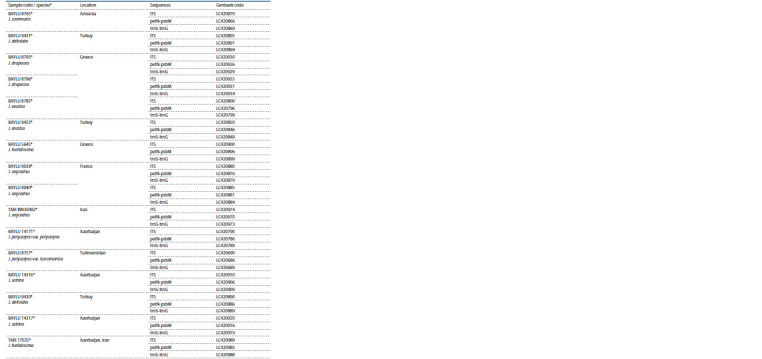
Table 2end * The samples taken for comparison from (Hojjati et al., 2018), their species are indicated.

Haplotype diversity (HD), nucleotide diversity (π), and the number of nucleotide substitutions (M) were calculated for each species using DnaSP 6.0 (Rozas et al., 2017).

The relationships between haplotypes of sequences from three marker sites were reconstructed by the TCS method implemented in the PopArt program (Bandelt et al., 1999).

## Results and discussion

In 2014, R.P. Adams (2014a) developed and published a determinant key for J. deltoides, which makes it possible to distinguish individuals of this species from J. oxycedrus. According to the determinant, the maximum needle length of J. deltoides is less than that of J. oxycedrus and equals 13.0 mm (for J. oxycedrus it is 15.0 mm). The length of the needles of the Crimean population of J. deltoides is 12.94 ± 0.19 mm, which corresponds to the dimensions declared by Adams. At the same time, a significant number of individuals were found (in the vicinity of the city of Inkerman and the village of Kudrino; on the mountains of Kara-Dag, Koba-Kaya, Dragon, on the rocks of Kullu-Kaya and on Cape Martyan), the length of the needles of which is from 18 to 20 mm. These individuals aroused the greatest interest for further research. Needle width, according to Adams (2014a), is also a defining feature. Under the conditions of the Crimea, this indicator is 1.39 ± 0.02 mm, which corresponds to the western group of junipers, namely J. oxycedrus.

**Fig. 2.**
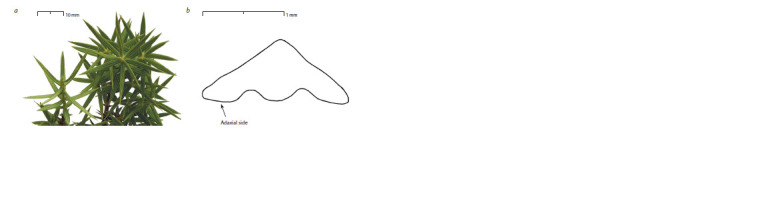
Needles of the Crimean cryptic population (localization – mt. Tolaka-Bair): a, general view; b, a schematic representation of the curvature of the adaxial surface of the needles.

The needles had no differences in color and shape of the base. All needles are light green with a deltoid base, which is typical for J. deltoides (Fig. 2, a). At the same time, the cross section of the needles showed that a significant part of the individuals (34 %) are characterized by the curvature of the adaxial surface of the needles (see Fig. 2, b). According to Adams (2014a), this is a distinctive feature of J. oxycedrus. The length of the needles with this type of stomatal bands is 11.87 ± 0.24 mm. Thus, it was found that the morphological features of the needles of the Crimean population of J. deltoides simultaneously exhibit signs of both J. deltoides and J. oxycedrus. On the basis of the identified features of the vegetative organs, it is not possible to attribute individuals to one of the species

The second distinctive morphological feature of junipers are cones. In the case of the cryptic pair, J. deltoides/J. oxycedrus, the main role in determining the species is played by the inosculation of cone scale tips, and to a lesser extent, by the size and color of the cone of cones, the presence or absence of plaque on them.

**Fig. 3.**
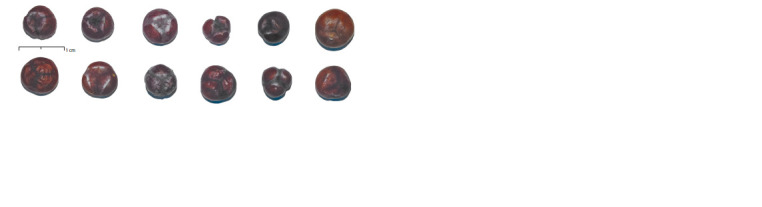
Morphological heterogeneity of cones of the cryptic population of Crimea.

The cones of the Crimean cryptic population have a significant number of morphological variants (Fig. 3). At the same time, the coloration is almost the same for all of them, and the smoke-blue coating appears to varying degrees, regardless of the shape of the cones. The shape, in turn, differs very much (from spherical to triangular). There are individuals with cones, the covering cone scale tips of which are visually indistinguishable and their tops are completely fused. A similar phenomenon is characteristic of J. oxycedrus. At the same time, the needles of these individuals are defined as the needles of J. deltoides. The second type of cones is almost triangular due to clearly visible three covering cone scale tips (characteristic of J. deltoides). The needles of these individuals show signs of both species. To a greater extent, there are intermediate variants, in which the bases of the cone scale tips grow together, and their tops move away from each other to varying degrees.

The size of the cones turned out to be the most stable trait and differed within the error on all test areas. The average sizes of cones are: d1 (conditional height) is 7.54 ± 0.14 mm, and d2 (conditional width) is 9.11 ± 0.09 mm, which is more consistent with the Turkish population of J. deltoides.

Thus, the morphological analysis of the vegetative and generative organs of J. deltoides showed that when these two characters are combined; it is not possible to reliably distinguish between individuals of J. deltoides and individuals of J. oxycedrus. J. oxycedrus is a basal species, while J. deltoides is a cryptic one. As a result, the issue of conducting phylogenetic studies is especially acute for determining the systematic belonging of the Crimean population to one of the species.

For phylogenetic analysis, the nucleotide sequences of three marker sites (ITS, petN-psbM and trnS-trnG) of 16 Crimean samples and 17 samples from the work (Hojjati et al., 2018) were used (see Table 2)

Phylogenetic trees constructed from individual marker sequences are presented in Supplementary Materials 1–31. The use of ITS and petN-psbM marker sequences allowed us to obtain topologies where each species forms a separate clade and their phylogenetic definition is unambiguous. The topology obtained by analyzing the trnS-trnG sequences does not allow separating the species of J. communis and J. del-toids.

Supplementary Materials are available in the online version of the paper: http://vavilov.elpub.ru/jour/manager/files/Suppl_Lantushenko_Engl_27_4.pdf


**Fig. 4.**
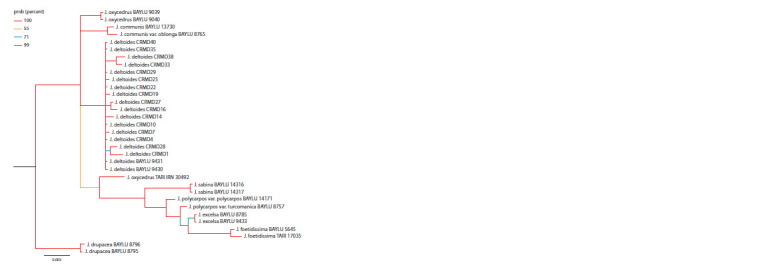
A phylogenetic tree constructed by the Bayes method based on a combined sequence including nuclear (ITS) and chloroplast (petN-psbM, trnS-trnG) non-coding regions of the genome. Node support values are shown in color.

A phylogenetic tree was also constructed taking into account all the nucleotide fragments studied in this work (Fig. 4). It can be seen that the samples of each species form clades with high (more than 75 %) support. The exception is the specimen J. oxycedrus TARI IRN 30492, for which the phylogenetic definition is not unambiguous: according to the sequences trnS-trnG and petN-psbM, it forms a clade with samples of the species J. deltoids, and according to the fragment of ITS and the analysis of the combined sequences, it forms a clade with species of the section Sabina. All the samples from the Crimea studied in this work formed a clade with sequences of the species J. deltoides 9430 and 9431 (BAYLU) growing in Turkey. In this clade, three pairs of samples (CRMD1 and CRMD28, CRMD16 and CRMD27, CRMD33 and CRMD38) form separate branches with high support, but there is no correlation between them by phenotypes and geographical location.

**Fig. 5.**
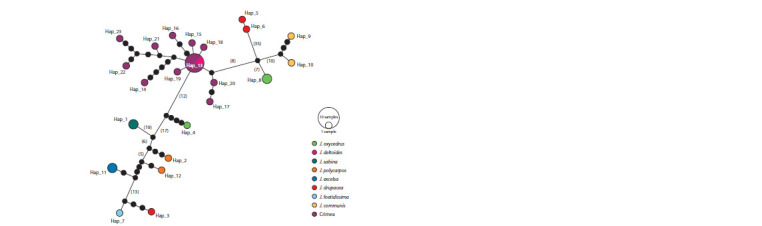
Haplotypic network constructed by the TCS method. The color indicates belonging to the species, the size of the circle is the number of samples in the haplotype, small distances are represented as dots (1 point – 1 replacement), for large ones the number of substitutions is given in brackets.

The haplotypic network constructed from the nuclear and chloroplast regions of the genome for the samples listed in Table 2 is shown in Figure 5. It can be seen that the Crimean population of J. deltoides is characterized by a quite large number of haplotypes: 11.

In the previously studied populations of juniper trees of other species (J. excelsa, J. polycarpos, and J. foetidissima) on the northern border of the distribution area, in the Crimea, the Caucasus and Dagestan (Sadykova et al., 2021), much smaller haplotypic diversity was found: sequences of 17 J. excelsa samples form two haplotypes, 16 J. foetidissima samples form four haplotypes, 15 samples of J. polycarpos – one haplotype

The genetic variability of nuclear and chloroplast regions of genes was analyzed. The analysis of the parameters presented in Table 3 allows us to conclude that the greatest variability is characteristic of the ITS nuclear fragment, and the least variability is characteristic of the trnS-trnG fragment. The greatest variability of the nuclear sites of marker nucleotide sequences is characteristic of other juniper species (Mao et al., 2010; Hojjati et al., 2018).

**Table 3.**
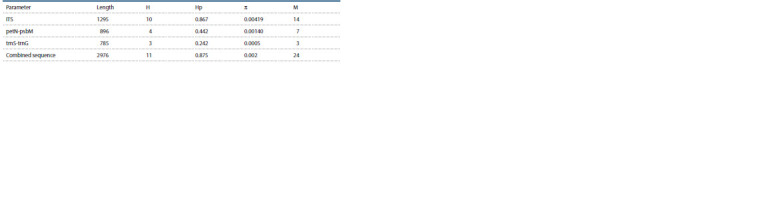
Characteristics of the nucleotide sequences studied in this work Notе. H is the number of haplotypes, Hp is the haplotypic diversity, π is the nucleotide diversity, M is the number of mutations.

It follows from Figure 5 that the nucleotide sequences of CRMD4, CRMD10, CRMD22, CRMD29, CRMD35, CRMD40 samples formed a common haplotype with the sequences BAYLU:9430 and BAYLU:9431.

The sequences obtained in this work formed a clade with J. deltoides 9430 and 9431 (BAYLY) specimens growing in Turkey (Table 4). Thus, the analysis carried out in this work allows us to attribute the studied samples to the species J. deltoides

**Table 4.**
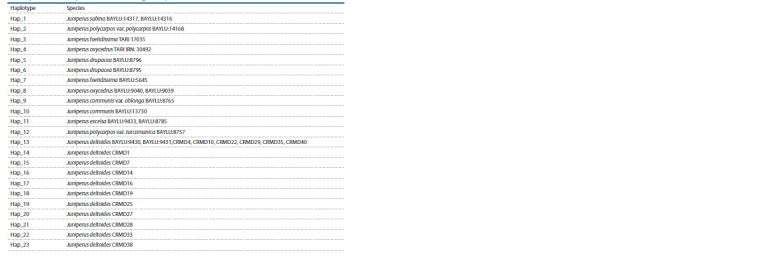
List of haplotypes of nucleotide sequences of samples of the Crimean population and samples taken for comparison from the work (Hojjati et al., 2018)

## Conclusion

Based on the studies of vegetative organs, it was found that the length of the needles of individuals of the Crimean cryptic population is 12.94 ± 0.19 mm, which is typical for this species. At the same time, there are individuals with needles of much greater length (18–20 mm). The cross section of the needles, regardless of its length, in 34 % of cases shows signs of J. oxycedrus, expressed in the curvature of its adaxial surface.

Cones manifest significant morphological heterogeneity. Their shape varies from spherical to triangular, depending on the degree of fusion of the covering cone scale tips. At the same time, it was found that the same individuals can simultaneously show signs of J. deltoides (by vegetative organs) and signs of J. oxycedrus (by generative organs).

Thus, we can conclude that these morphological features are not reliable for determining the systematic affiliation of individuals. Thus, the only possible way to determine it is to conduct genetic research.

Phylogenetic analysis has shown that nuclear regions of genes have greater variability than chloroplast ones. The sequences obtained in this work for the Crimean population formed a clade with samples of J. deltoides 9430 and 9431 (BAYLU) growing in Turkey, which makes it possible to attribute the studied samples to the species J. deltoides.

## Conflict of interest

The authors declare no conflict of interest.
